# Addressing unmet needs of adolescents to prevent unintended pregnancy in the Asia Pacific region

**DOI:** 10.1016/j.lansea.2025.100693

**Published:** 2025-11-10

**Authors:** Sumita Banerjee, Bunyarit Sukrat, Ketkesone Phrasisombath, Marijuzca Y. Nicolas, Bibek Kumar Lal, Sadia Rahman, Marie Habito, Elissa Kennedy, Stephen Bell

**Affiliations:** a FP2030 Asia Pacific Regional Hub, Kuala Lumpur, Malaysia; b Ministry of Public Health, Government of Thailand, Thailand; c Ministry of Health, Government of Lao PDR, Lao PDR; d Department of Health, Government of the Philippines, the Philippines; e Ministry of Health and Population, Government of Nepal, Nepal; f Burnet Institute, Melbourne, Australia; g MCRI, Melbourne, Australia; h School of Public Health and Preventative Medicine, Monash University, Melbourne, Australia; i School of Population and Global Health, University of Melbourne, Melbourne, Australia

The World Health Organization (WHO) recently published an updated guideline on preventing early pregnancy in low- and middle income countries.^1^ This is an urgent reminder that adolescent pregnancy remains a global public health concern with serious and lasting consequences for young people, and that continued investment in adolescent pregnancy prevention will create healthier, more equitable societies where young people can thrive, now and into the future.^1^

Adolescent pregnancy remains an urgent regional priority across the Asia Pacific region, where over 8 million pregnancies occur annually among girls aged 15–19.^2^ Almost half are unintended, resulting in 2 million abortions and 750,000 mistimed or unwanted births.^2^ Complications related to pregnancy and childbirth remain among the leading causes of death of girls aged 15–19 years in Asia and the Pacific,^3^ while socio-economic outcomes resulting from adolescent pregnancy (school drop-out, constrained livelihoods, early marriage) perpetuate poverty and gender inequality across the life course and generations.^4^^,^^5^ The UN Sustainable Development Goals related to maternal and child health, education, gender equality and poverty will not be reached without reducing unintended adolescent pregnancy.^6^

There is substantial economic benefit of reducing adolescent pregnancy. An additional $5 billion to scale up proven interventions for girls by 2030 would prevent 1.4 million unintended pregnancies and 1.1 million child marriages, generating $13.4 billion in economic benefits by 2050.^7^ Moderate investment is particularly important in the Asia Pacific region, which is home to nearly one billion adolescents aged 10–24, or 60% of the world's young people.^8^ In a region grappling with an ageing population, the economic impacts of unintended pregnancy during adolescence will be profound, limiting girls' labour force participation and economic productivity.

In response to growing unmet needs among young people in the region, the FP2030 Asia Pacific Regional Hub^j^ held regional meetings—in Kathmandu, co-organized with the Ministry of Health and Population, Government of Nepal (November 2024); in Bali, co-organised with the Ministry of Population and Family Development and Ministry of the State Secretariat, Government of Indonesia, and UNFPA Indonesia (October 2025)—with dedicated sessions on the prevention and care of adolescent pregnancy. Each of these meetings brought together over 70 participants from 13 countries to share learnings, promote policy dialogue and catalyse regional action and collaboration with a spotlight on this critical issue. The Bali meeting included the formation of a regional working group to tackle issues of adolescent pregnancy across the region.

Panel 1 highlights case studies from across the region, in Lao PDR, Thailand, the Philippines and Nepal. In settings where adolescent pregnancy is shaped by a complex interplay of socio-cultural norms, limited access to SRH information and services, and systemic gender inequalities, these case studies illustrate data, national successes and priority next steps in supporting young people with access to family planning to prevent pregnancy. These case studies also point to the importance of: (i) policy levers established through strategic commitments by ministries of health to deliver inclusive adolescent-responsive services, expand access to family planning to unmarried young people, and improve data collection to support evidence-based decision making; and (ii) multi-sectoral action across diverse ministries to enhance SRH education and awareness in school, community and digital settings.

Panel 1 **Adolescent pregnancy prevention strategies in Lao PDR, Nepal, the Philippines and Thailand.**

**Table:** 

Country, context and data	What's working?	Next steps
**Lao PDR**: Adolescent birth is 89 per 1000 women aged 15–19 years. Between 2010 and 20, the adolescent birth rate remained high, particularly in rural areas and particular provinces. Nearly 25% of girls aged 15–19 are married or in a relationship; 19% of women aged 20–24 have given birth before turning 18. Conservative norms stigmatise sex outside union, accept early marriage (including as a response to pregnancy), and constrain adolescent girls' decision-making about contraception. Barriers accessing adolescent-responsive reproductive health services and limited access to contraception contribute to high unmet need for family planning.	The National Adolescent and Youth Health Strategy (2021–30) promotes a cross-governmental approach to reduce adolescent pregnancies. The Ministry of Health focuses on strengthening adolescent-friendly health services, ensuring confidential access to contraception and skilled birth attendance. The Ministry of Education implements comprehensive sexuality education in schools. Other programs include expanded access to adolescent-friendly services to eight provinces, including the offer of family planning to married and unmarried youth; legal measures to prevent child marriage; awareness campaigns to engage families and communities to reduce stigma and promote informed decision-making among adolescents.	Strengthening multisectoral coordination to prioritise adolescent family planning; meaningful youth participation in decisions; expanding CSE in and out of schools; improved access to adolescent-friendly SRH services through provider training and private sector engagement; gender-transformative approaches to encourage active fatherhood and male responsibility; partnerships with youth organisations, civil society organisations and community groups to sustain advocacy, mobilize resources, and scale up successful models.
**Thailand:** Adolescent birth rate increased from 31.1 to 53.4 per 1000 women aged 15–19 years (2000–2012) and has since fallen to 26. By 2026, Thailand hopes to reduce the adolescent birth rate to 15. In 2022, 68% of married 15–19 year-olds were using a modern method; 87% had demand for family planning satisfied with modern methods. There is a lack of systematically collected data for unmarried sexually active adolescents. Adolescent pregnancy is more strongly associated with socioeconomic status, education, certain ethnic minorities and socially marginalised populations.	In 2016, Thailand established the Prevention and Solution of the Adolescent Pregnancy Problem Act, B.E. 2559. This outlined the rights of adolescents to information, knowledge and reproductive health services, confidentiality, and to make their own decisions. It involved multisectoral collaboration and action across six ministries, with a focus on education, the role of families and communities, youth-centred health services, systems of social support, and knowledge management. The government recently introduced “Strong Community for Healthy Sexuality” to mobilise community action to prevent adolescent pregnancies and support adolescent wellbeing.	Strengthened implementation of the Act; male engagement initiatives to address harmful gender norms; youth-led advocacy and contribute to policy and program development; expansion of adolescent-friendly SRH services; targeted advocacy campaigns to raise awareness and reduce stigma surrounding adolescent sexual and reproductive health.
**Nepal:** The Nepal Demographic and Health Survey 2022 indicated 14% women aged 15–19 years had been pregnant (10% gave birth; 2% experienced pregnancy loss) and low use of family planning among adolescents. Early marriage, gender inequality, poverty, and social expectations contribute to high adolescent birth rates; stigma prevents pregnant adolescents from continuing education.	Nepal has taken key steps to address this issue: setting the legal marriage age at 20 under the Reproductive Health Act; developing adolescent-friendly health service guidelines; legalising safe abortion; expanding access to modern contraceptives; integrating comprehensive sexuality education aligned with international standards.	Enhanced delivery of CSE aligned with international guidelines; expanded digital interventions to overcome geographic and social barriers.
**The Philippines:** The Field Health Services Information System indicates the adolescent birth rate increasing from 15 in 2019 to 25/1000 in 2023; 9/17 regions had rates above the national average. Unintended pregnancy occurs in contexts where young women are unaware of contraceptive options, modern contraceptives are perceived as being for married women, and family experiences of poverty lead to school drop-out.	In 2021, Executive Order No. 141 declared adolescent pregnancy a national priority, promoting a multisectoral approach. Led by the Commission on Population and Development, an interagency council was created with representatives from different agencies (including youth groups and health, education and social welfare sectors) to address child and adolescent pregnancy holistically, recognising health, socio-economic and cultural influences.	Halt increased adolescent fertility through a Comprehensive Action Plan aligned with EO No. 141 through: Comprehensive Reproductive Health Education; expanded access to quality reproductive health services; strengthened measures against sexual abuse, coercion, and gender-based violence; socio-economic development and social protection for adolescents and young parents; child and youth participation to ensure programs are responsive to their needs.

Future progress towards equitable, voluntary, rights-based family planning to prevent unintended adolescent pregnancy across Asia and the Pacific would be advanced by continued investment and funding in four priority areas. First, strengthening comprehensive sexuality education (CSE) requires designing curricula that aligns with international CSE standards (e.g. explicitly addressing restrictive gender and social norms that perpetuate early unintended pregnancy cycles), as well as overcoming structural implementation barriers to reach young people in community settings. This includes developing community-led approaches that engage families and traditional leaders as allies and reach young people who are not in school, while ensuring youth co-design of curricula and information, education and communication messaging that reflects the diverse lived realities of adolescents living in rural and urban settings, as well as from ethnic minorities and socially disadvantaged backgrounds.

Second, addressing legal, policy and health systems barriers should extend beyond policy reform to implementation of services and programs that are available and accountable to young people and their communities. This includes reducing financial barriers to contraceptive access, tackling community stigma around premarital sex and addressing provider bias through training and accountability mechanisms. Multi-sectoral coordination that meaningfully engages adolescents in policy development would enable young people to shape policies directly affecting their lives, while integrating intersectionality frameworks to address how age, gender, household location, marital status, ethnicity and socioeconomic status create systemic, compounded access barriers.

Third, scaling up married and unmarried young people's access to safe, modern contraceptives through public, private and non-governmental service provision could embrace WHO-endorsed self-care approaches that can be provided within and beyond formal health services.^9^ Self-care strategies are particularly valuable where young people's formal healthcare access is limited or stigmatised. Innovation around contraceptive care for young people might include: community-based distribution models that leverage peer networks and youth leadership; task-shifting of contraceptive care to community health workers and other community-based health professionals; equitable, safe digital technologies; and developing sustainable supply chains that address geographic and economic barriers in rural, peri-urban and urban communities where adolescent pregnancy rates remain highest.

Fourth, enhanced data can ensure interventions are targeted and effective, and based on current understandings of unmet need. We propose the use of youth-led qualitative research to centre young people's experiences and expertise; geolocated survey data to identify priority hotspots for improved service access in areas of unmet need; co-design and piloting of novel strategies that enhance young people's safe access to modern contraceptives; and robust modelling to support governments and other partners with decision-making around cost-effectiveness, return on investment and resource optimization. Ongoing monitoring research should disaggregate data by intersecting identities and be guided by indicators of success that reflect adolescents' priorities and community perspectives on health equity and justice.

Critically, all four priority areas should also be responsive to new and emerging threats associated with climate change, including displacement, food insecurity, extreme weather events, and climate-related mental health impacts that disproportionately affect adolescent girls and compound existing vulnerabilities to unintended pregnancy.

## Contributors

All authors contributed equally to conceptualising, writing, reviewing and editing this work.

## Declaration of interests

We declare no competing interests.

## Footnotes

j See the following website for more information about FP2030's Asia Pacific Regional Hub: https://www.fp2030.org/regional-hubs/asia-and-pacific/.
